# Effect of the Dynamic Porous Structure Generation in Laser Irradiated Multi-Functional Coatings

**DOI:** 10.3390/ma17184493

**Published:** 2024-09-13

**Authors:** Wenzhi Li, Yichao Zhu, Zhiping He, Lihong Gao, Zhuang Ma

**Affiliations:** 1 China Helicopter Research and Development Institute, Jingdezhen 333001, China; 2 School of Materials Science and Engineering, Beijing Institute of Technology, Beijing 100081, China

**Keywords:** high reflection, reaction energy dissipation, skeleton porous structure, multi-functional laser protection

## Abstract

Focusing on solving the adverse laser-inducing damage problem, high-power laser-resistant strategies have attracted more attention. In order to improve the laser-resistant property, a novel dynamic porous structure generation idea for laser irradiation was presented in this study, both of high-reflection and reaction endothermic effects. A detailed investigation on phase structure change, optical properties variation, micro-structure evolution, and substrate temperature development during laser irradiation was performed. The initial reflectivity of two coatings at 1064 nm was high, around 80–90%. During laser irradiation, the reflectivity grew continuously, reaching a maximum of 93%. During laser irradiation, a skeleton porous structure formed, promoted by the endothermic reaction of aluminum tri-hydroxide, whose structure contributes to the heat insulation from surface to interior. Thus, the prepared coating showed excellent anti-laser ablation performance, being dependent on its thermal insulation by the reaction-generated porous structure; high reflectivity by surface; and heat dissipation by endothermic reaction. Under 2000 W/cm^2^, 10 s laser irradiation (spot area is 10 mm × 10 mm), the back-surface temperature is just 159 °C, which is far away from the melting point of aluminum substrate. The coatings and strategy mentioned in this study have a great potential to be applied in the anti-laser field.

## 1. Introduction

With the widespread application of laser technique and rapid development of laser power, damage induced by high-power lasers has become a serious problem [1,2,3,4]. Many researchers have realized the necessity of achieving laser resistance [5,6,7]. According to the mechanism of laser interaction with matter, the energy transformation and deposition of incident high-power laser induces the final damage on substrate [8]. Thus, temperature increasing caused by huge laser energy deposition is the key reason for laser destruction, since laser irradiation could induce a thousand-degree-centigrade temperature increase in a few seconds [9,10,11]. So, timely heat dissipation is an important solution for laser resistance. Considering that a laser is a kind of light and interacts with matter by photons, reducing the number of photons deposited to material could solve the laser destruction threat radically. Therefore, a protective layer with high reflection, which could decrease the absorption of the laser energy and delay the onset of obvious laser destruction, was put forward with no doubt [12]. However, no matter how the reflection is, the absorption of laser energy cannot be avoided. Then, thermal insulation and heat dissipation are also necessary. 

In order to explore the better protection strategies under laser irradiation, many anti-laser materials have been developed in recent years. Organic–inorganic composites adding fillers with high reflectivity and low thermal conductivity show good laser resistance [13,14,15,16,17]. However, a low burning point and oxidation problems make these coatings only applicable under low laser power (<1000 W/cm^2^, 10 mm^2^ spot, power < 1000 W). Furthermore, the high reflectivity of the filler is hard to be exhibited due to the low-reflection influence of matrix. The laser ablation properties of pure inorganic materials, such as ceramic, is also investigated. Plasma-sprayed La_1−x_Sr_x_TiO_3+δ_ coating exhibited high reflectivity during laser irradiation, but under 1000 W/cm^2^ (10 mm^2^ spot, power 1000 W), the coatings were destroyed rapidly due to the heat concentration [9]. A graphite–SiO_2_ composite shows a different laser ablation mechanism by carbothermal reaction, and its optimized structure could resist higher laser power [18]. An ultra-high temperature ceramic-filled ZrO_2_ sol–gel strategy provides a new idea for laser protection coatings. It can bear 6.3 kW/cm^2^ (0.3 mm circular spot, power about 600 W) of laser irradiation [19]. Since there are many reworks reporting the progress of laser-ablation-resistant materials, all the related works focus on incident energy insulation or dissipation strategy design and its characterization, instead of exploring the protective limitation.

Among the heat insulation strategies, porous structure is a typical ultra-low thermal conductivity structure, which could obstruct most energy transferred to the substrate, offering protection. Thus, a gas generation composition is needed in order to promote the generation of porous structure. As a widely used flame retardant, aluminum tri-hydroxide (ATH) is a good candidate filler choice; it starts to decompose at low temperature, about 180–220 °C [20]. Additionally, ATH has high heat endothermic enthalpy, shown in Equation (1) [21]. The decomposition process of ATH could cool down the heated material surface and produce a protective layer of Al_2_O_3_ with good high temperature stability. Furthermore, the decomposition reaction product, Al_2_O_3_, as a common infrared reflection material, has relatively high reflectivity compared with ATH [22,23].

2Al(OH)_3_→Al_2_O_3_ + 3H_2_O (△H = 1050 J/g) (1)

Considering that the coating applies in harsh conditions, especially ultra-high temperature, an inorganic binder with a good, high-temperature performance is a better choice. During the application, the inorganic binder plays an important role because it has to sustain the high laser energy and carry the filler at the same time. Since the formed reticular Si–O structure of sodium silicate matrix after curing has good high-temperature resistance, it could fix the filler stability and improve the thermal stability of the matrix, and it could also be used as the binder material [24]. However, laser energy will still be deposited to the interior material. This part of the absorbed energy is given to retardance at the initial stage; thus, two routes are reliable: heat isolation by low thermal conductivity or heat diffusion by high thermal conductivity. ZrO_2_ exhibits excellent properties, such as high reflectance, low thermal conductivity, high thermal shock resistance, high-temperature phase stability and chemical durability, which meet the requirements of the former route [25,26,27]. BN has high thermal conductivity (higher than 70 W/m·K) and excellent high temperature resistance [28], which could enhance the thermal diffusion of the whole coating.

In this paper, pure inorganic coating under laser irradiation was investigated. Sodium silicate was used as binder and cured at high temperature. ATH, with cooling characteristics and excellent potential property of reaction production, was selected as a gas generator filler of coating. Additive thermal diffusion functional fillers were chosen as ZrO_2_ and BN. A comparison analysis of prepared coatings before and after continuous-wave laser ablation was conducted. The evolution of critical performances during laser irradiation was studied, including the reflection, forward-scattered light, and back-surface temperature. Finally, the laser ablation behavior was discussed, and the anti-laser property of prepared coating was evaluated as well.

## 2. Materials and Methods

An aluminum substrate with the size of Φ25 mm × 2 mm was used as the substrate. In order to remove the surface oxide layer, a grit-blasting technique was implemented. Commercial analytically pure Al(OH)_3_ (Alfa Ascar, Ward Hill, MA, USA, particle size 10 μm), ZrO_2_ (Alfa Ascar, Ward Hill, MA, USA, particle size 5–10 μm), and BN (Alfa Ascar, Ward Hill, MA, USA, particle size 10–30 μm) powders were used as fillers. A sodium silicate (Spring Polymer, Zhuzhou, China) with a modulus of 2.7 and Baume degree of 48 was selected as binder. The raw materials were weighed according to the formulation listed in Table 1. Then, the mixture was stirred at 2000 rpm for 30 min to ensure the filler and binder could be mixed uniformly. Next, the coating was fabricated via a brushing method with the thickness of 1.5 mm using the commercially available coating machine (ZY-TB, Shandong Zhongyi Instrument Co., Ltd., Jinan, China). Finally, continuously gradient-curing, the prepared coatings were precured at room temperature for 24 h and then cured by the following continuous gradient-curing procedures: 50 °C for 2 h, 80 °C for 2 h, 110 °C for 2 h, 140 °C for 4 h, and then cooling with furnace. The heating rate is 5 °C/min.

The samples were irradiated by the high-power continuous laser (YSL-2000, IPG Photonics 1070 nm, Marlborough, MA, USA) with the spot area of 10 × 10 mm^2^. The variations of back-surface temperature and forward-scattered light during laser irradiation were record by the thermocouple and near-infrared (NIR) detector system (GD3561T, Xi’an, China). The laser ablation behavior was investigated at the laser density range from 1000 W/cm^2^ to 2000 W/cm^2^ and the irradiation time is 10 s.

The phase composition change in corresponding coatings before and after laser irradiation were characterized by X-ray diffraction (XRD, PANalytical Inc., X’Pert PRO MPD, Almelo, Holland). The XRD patterns were analyzed by JADE 10.0 software (Adobe Systems Software Ireland Ltd., Livermore, CA, USA). The surface and cross-section microstructure evolution were studied by scanning electron microscopy (SEM, HITACHI S4800, Tokyo, Japan). The chemical composition was characterized via energy-dispersive spectroscopy (EDS, Oxford Instruments Co., Ltd., Oxfordshire, UK). The reflectivity of the coatings were measured by an ultraviolet–visible–near-infrared (UV–VIS–NIR) spectrophotometer (Cray 5000, (Palo Alto, CA, USA). Meanwhile, the front-scattering light along the laser test was detected by a near-infrared (NIR) detector system (GD3561T, Zolix Instruments Co., Ltd., Beijing, China).

## 3. Results and Discussion

### 3.1. Macrostructure and Phase Analysis of Irradiated Coatings

**Figure 1:**
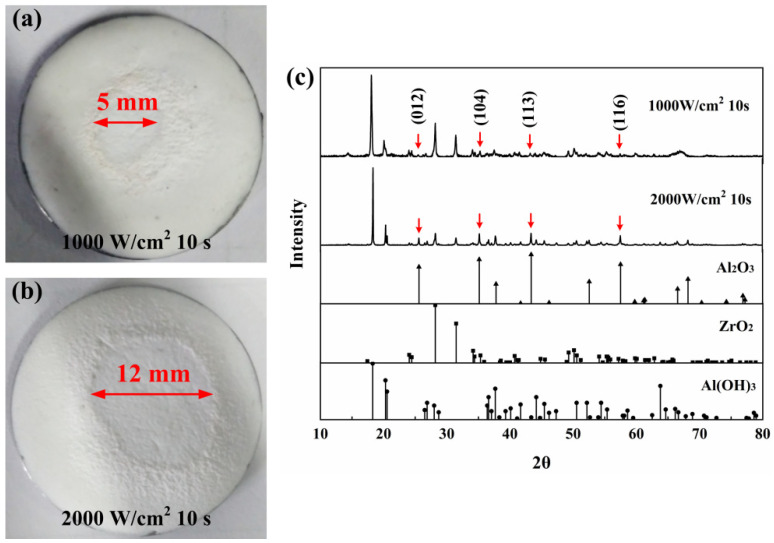
Macro-morphologies of AS-Z after laser irradiation: (**a**) 1000 W/cm^2^, 10 s; (**b**) 2000 W/cm^2^, 10 s, and (**c**) corresponding XRD patterns.

The surface macro-morphologies of AS-Z coatings after laser irradiation under different parameters are shown in Figure 1a,b, and the XRD patterns of each corresponding samples are individually shown in Figure 1c. A visible boundary between ablative reaction area and heat-affected area can be observed. It can be seen that with the laser energy increase, the size of ablative reaction area grew from 5 mm to approximately 12 mm, which is larger than the size of laser spot. The ablation area increased significantly, and the degree of ablation became aggravated. Because of the existence of the ZrO_2_ fillers, the heat diffusion would be blocked, so the expansion of ablation area was not very large at high laser power. Through the XRD patterns of the ablated AS-Z coatings shown in Figure 1c, with the laser power increase, the intensity of some main Al_2_O_3_ diffraction peaks, (012), (104), (113), and (116), appeared and became stronger. The appearance of the Al_2_O_3_ phase proves the happening of ATH endothermal composition reaction. Due to the occurrence of the decomposition reaction, the ablation area, especially the boundary, became rough.

**Figure 2:**
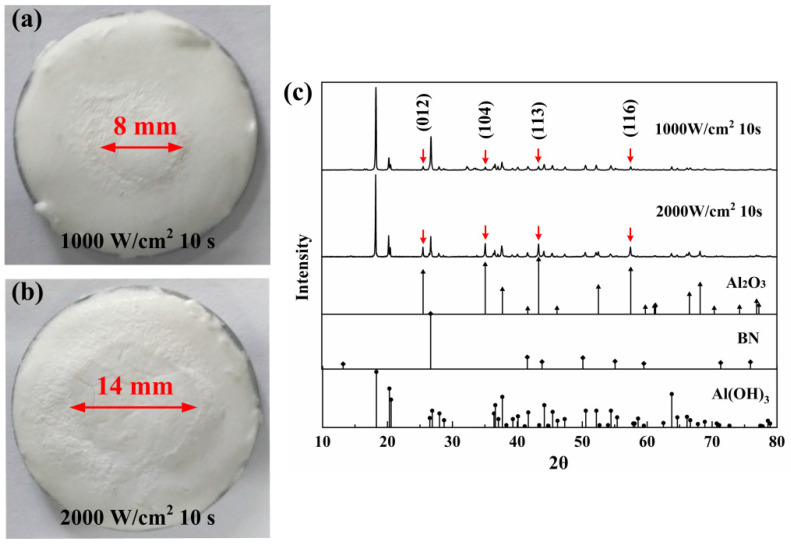
Macro-morphologies of AS-B after laser irradiation: (**a**) 1000 W/cm^2^, 10 s; (**b**) 2000 W/cm^2^, 10 s, and (**c**) corresponding XRD patterns.

Just like the phenomenon shown in ablated AS-Z coatings, the macro-morphologies and phase structures of the ablated AS-B coatings shown in Figure 2 were similar. However, the ablation area size of the AS-B coatings was larger than that of the AS-Z coating because of the addition of the high-conductivity phase BN, which improved the horizontal heat transfer and energy homogenization of surface. The ablation area rose from 8 mm to 14 mm. Besides the diffraction peaks of Al_2_O_3_, in these two coatings, all other diffraction peaks were same in the phase composition with raw materials. The fillers ZrO_2_ and BN all exhibited the single phase, so there is no phase transition which reveals the fillers were stable during laser irradiation. This phenomenon indicates that the energy provided by the incident laser could promote the occurrence of the reaction but was not high enough to induce the phase transition.

### 3.2. Optical Properties of As-Prepared and Irradiated Coatings

**Figure 3:**
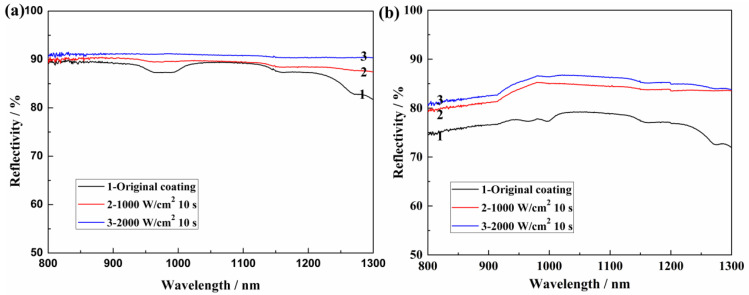
The optical reflectivity of as-prepared and laser irradiated coatings: (**a**) AS-Z coatings; (**b**) AS-B coatings.

The optical reflectivity at the range from 800 to 1300 nm of the as-prepared and laser-irradiated coatings are shown in Figure 3. It can be seen that all coatings performed good reflectivity at near-infrared wavelength, including the laser wavelength (1064 nm), which contributes to the good reflection of the fillers themselves. As shown in Figure 3a, AS-Z coatings exhibited the highest reflectivity values, all nearly 90%. While the reflectivity values of the AS-B coatings were a little lower than the AS-Z coatings (shown in Figure 3b), it was still higher than 75%. Especially at 1064 nm, the reflectivity after laser irradiation increased to 85%, which was similar to the reflectivity of the AS-Z coatings. These high values of reflection are comparable with metal materials, while their melting points are much higher than metal materials. These characteristics exhibit the excellent application potential of these coatings. Compared with the reflection data on each kind of coating individually, the reflectivity after laser irradiation was higher than that of the original coating, which was because of the generation of Al_2_O_3_. However, the reflection data of prepared coating is higher than that of the organic–inorganic composite, which is only around 60–70% [13,14,15,16,17].

### 3.3. Back-Surface Temperature and Front-Scattered Light Analysis

**Figure 4:**
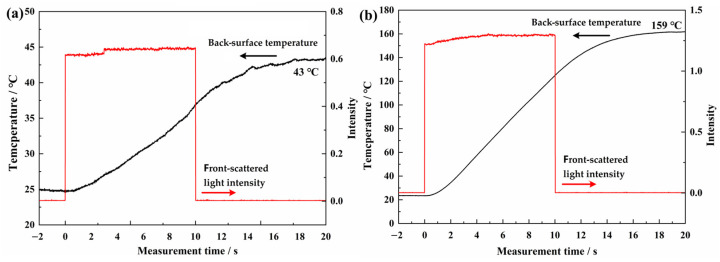
The back-surface temperature and front-scattered light of the AS-Z coatings. (**a**) 1000 W/cm^2^, 10 s; (**b**) 2000 W/cm^2^, 10 s.

**Figure 5:**
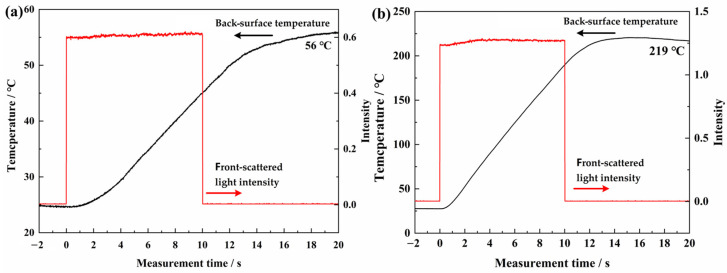
The back-surface temperature and front-scattered light of AS-B coatings. (**a**) 1000 W/cm^2^, 10 s; (**b**) 2000 W/cm^2^, 10 s.

During the laser irradiation, the back-surface temperature and front-scattered light were measured. It should be noted that the NIR detector only received one scattering signal from the front surface. The intensity of front-scattered light varies from one position to another, and it is also related to the incident laser power and coating-surface state. Therefore, the data obtained from different samples are not comparable. It could be used to evaluate the variation of optical reflection property of a single tested sample during laser irradiation. Moreover, the single front-scattered light could reflect the laser irradiation time. The results of the AS-Z and AS-B coatings under different laser parameters are shown in Figure 4 and Figure 5, respectively. From all the figures, it can be concluded that during the laser irradiation, the front-scattered light signal of coatings maintained stability, and the back-surface temperature rose continuously even after the laser irradiation was finished.

At 1000 W/cm^2^ laser irradiation, the front-scattered light intensity of both the AS-Z and AS-B coatings grew gradually. At 2000 W/cm^2^ for 10 s, the front-scattered light intensity increased at the beginning and maintained stability subsequently. The growth of front-scattered light intensity proves the increasing of optical reflection property. This phenomenon indicates that during the high-power laser irradiation, the coating surface state remained stable. Contributing to the occurrence of ATH endothermal reaction, part of the absorbed laser energy can be consumed. Together with the reflection improvement by generated Al_2_O_3_, incident laser energy can be dissipated further, so that the coatings were not penetrated. The back-surface temperature of two kinds of coatings grew steadily. The highest back-surface temperature of the Al substrate is just 219 °C, which is far away from the melting point of Al (650 °C). At each laser irradiation parameter, the highest back-temperatures of the AS-Z coatings (43 °C and 159 °C) were lower than those of AS-B coatings (56 °C and 219 °C). At 2000 W/cm^2^, the back-surface temperature growth rate of the AS-Z coating is 10.5 °C/s, which is just 2/3 of AS-B coating. Thus, thermal-insulation-type AS-Z coatings exhibited a better anti-laser ability according to the back-surface temperature. Honestly, the back-surface temperature of the prepared coating is much lower than the organic–inorganic composites, which is 424 °C at 1500 W/cm^2^ for 10 s [29].

### 3.4. Evolution of Micro-Morphologies during Laser Irradiation 

Since the laser interacted directly with the exposed coating structure, besides the contribution of ATH-decomposing energy consumption and reflection improvement by the generated Al_2_O_3_, the ablated micro-structure must play the key role in the laser-resistant process. From the evolution analysis of the ablated coating structure, the laser influence in coating and the laser ablation behaviors of coating can be explored in depth.

**Figure 6:**
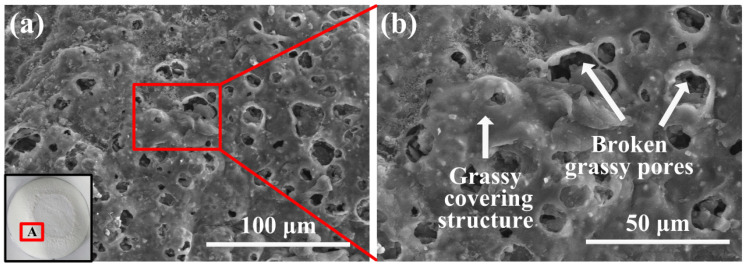
(**a**) Micro-surface morphology and (**b**) corresponding magnified image of the heat-affected area (area A) of the laser-ablated AS-Z coating.

The samples under 2000 W/cm^2^ for 10 s exhibited the worst ablation condition, so we analyzed these samples in detail. The laser irradiation area of each ablated coating can be divided into two parts: (a) the central area—ablation area and (b) the boundary area—heat-affected area. For the thermal-insulation-type AS-Z coatings, Figure 6a shows the micro-structure of the heat-affected area, which is located in area A of the coating (shown in the low left corner of Figure 6a). From the magnified surface morphology shown in Figure 6b, a grassy covering structure and broken grassy pores can be observed. In this area, the continuous sodium silicate surface layer melted and became a viscous liquid. Under the favor of the H_2_O gas generated by ATH decomposition, many glassy bubbles formed, and then the formed bubbles were broken by the interior H_2_O gas scouring. These grassy pores would contribute to accelerating the interior gas escape and forming the gas diffusion channels gradually.

**Figure 7:**
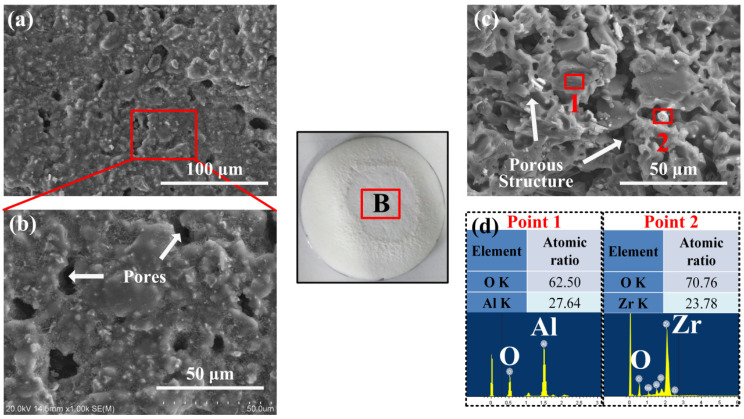
(**a**) Micro-surface morphology and (**b**) corresponding magnified image; (**c**) cross-section morphology and (**d**) related point 1 and 2’s EDS test of the ablation area (area B) of the laser-ablated AS-Z coating.

Figure 7a presents the surface morphology of the ablation area of the AS-Z coating (area B). This area is directly contacted by the laser, so this area suffered the worst laser ablation environment. From the magnified surface morphology, there is something different in the morphology of the heat-affected area: the grassy structure disappeared. Only the pores with the size of several microns formed by gas scouring were left. The surface exhibits a crystal-like structure. More important is that the coating reveals a good mechanical property. Even in this harsh laser ablation condition, no cracks appear. The cross-section morphology shown in Figure 7c gives the explanation of its good laser ablation property. Due to the initial thermal insulation effect of ZrO_2_ particle, the temperature increase in the irradiation coating was accelerated. Soon, the temperature reached the melting, evaporation temperature of sodium silicate and reaction temperature of ATH. When the sodium silicate vanished and the ATH reacted within a several-hundred-microns depth range from the irradiation surface, a porous structure was formed. Based on the EDS analysis shown in Figure 7d, it can be concluded that this porous structure consisted of Al_2_O_3_ skeleton and embedded ZrO_2_ filler. The energy consumption of ATH endothermal reaction and the stress-release function of the formed porous structure promote the maintenance of the integrity of the irradiated coating. Moreover, this porous structure contributes to gaining a low thermal conductivity and preventing the heat transfer to the inside.

**Figure 8:**
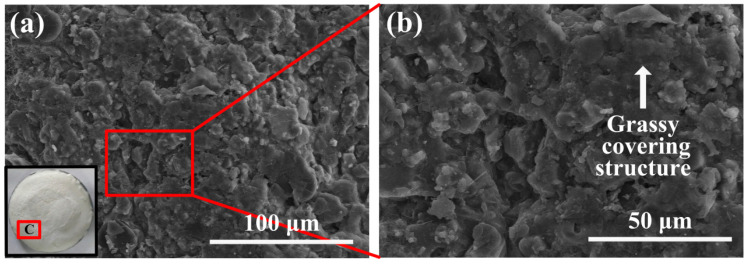
(**a**) Micro-morphologies and (**b**) corresponding magnified image of the heat-affected area (area C) of the laser-ablated AS-B coating.

The heat-affected area surface morphologies of the AS-B coatings are totally different from the AS-Z coatings. As shown in Figure 8, at the heat-affected area of the AS-B coating, most of the sodium silicate vanished because this coating suffered from a more serious heating environment, which can be proven by the back-surface temperature analysis. Since the back-surface temperature of the AS-B coating under 2000 W/cm^2^ for 10 s is 60 °C higher than that of the AS-Z coating, the front-surface temperature of the AS-B coating is also much higher. Therefore, only a glassy-like structure covered part of the surface and formed a compact crystal structure. Only a few pores appeared.

**Figure 9:**
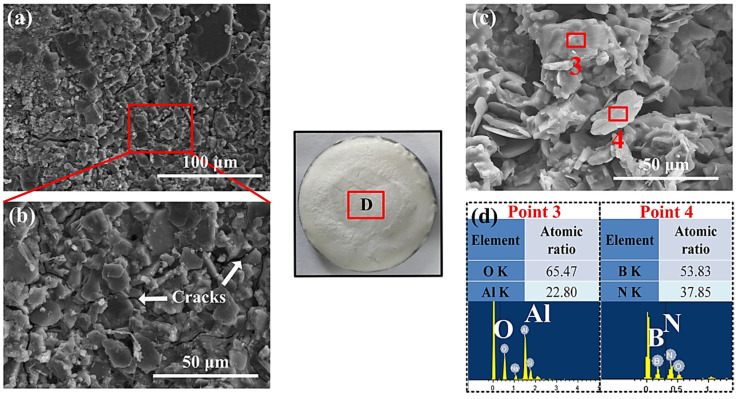
(**a**) Micro-surface morphology and (**b**) corresponding magnified image; (**c**) cross-section morphology and (**d**) related point 3 and 4’s EDS test of the ablation area (area D) of the laser-ablated AS-B coating.

At the central ablation surface area shown in Figure 9a,b, it can be seen that the crystal particles stacked with each other. Because of the high temperature sintering, this crystal-like particle sintered together and formed a crack structure. From the cross-section morphology of the AS-B coating, we can see that flake BN exhibited irregular distribution. Due to the particle stacking of BN, the heat conduction in both horizontal and vertical direction is fast. This is the reason why the AS-B coating had a higher back-surface temperature than the AS-Z coatings and suffered in more harsh thermal conditions. Therefore, few porous structures were generated.

The ablation behaviors and mechanisms of the prepared ATH coatings indicate that the coating that added the low-thermal-conductivity filler will have a good laser ablation resistance under high-laser-power irradiation, which is caused by its thermal insulation by the reaction-generated porous structure, high reflectivity by the surface, and heat dissipation by the endothermic reaction. This material and its protection strategy have great application potential as high-power-ablated surfaces to further enhance their ablation resistance.

## 4. Conclusions

ATH–sodium-silicate coatings added with ZrO_2_ or BN are fabricated and irradiated using a high-power continuous laser. Their initial reflectivity at corresponding laser wavelength is similar, around 80–90%. During laser irradiation, their reflectivity grew continuously, up to about 93%. Due to the energy consumption of ATH decomposition and their high reflectivity, the back-surface temperatures of these two coatings were both much lower than the destruction point of the substrate. Under the synergistic effect of binder and generated gas of the AS-Z coating, porous Al_2_O_3_ skeleton structure was generated, which blocked a huge energy transfer to the substrate and reduced the substrate temperature a lot. However, because of the lower reflectivity, irregular distribution of BN and generated cracks, the AS-B suffered from a more serious laser-heating condition, which promoted the sintering of AS-B coating. Additionally, with the formation of the porous Al_2_O_3_ skeleton structure in the AS-Z coating, on the one hand, the thermal stress of the coating could be reduced extremely; on the other hand, more heat could be insulated. Thus, the AS-Z coating shows multi-laser-protective mechanisms combined with thermal insulation, high reflectivity, and heat dissipation. It illuminates a better laser-ablation-resistant property, which has the great potential for application in an anti-laser area.

## Figures and Tables

**Table 1:** The formulation of coatings.

Sample	Sodium Silicate/wt.%	ATH/wt.%	ZrO_2_/wt.%	BN/wt.%
AS-Z	50	40	10	0
AS-B	50	40	0	10

## Data Availability

The data are contained within the article.
